# Novel calibration approach for particle size analysis of microplastics by laser ablation single particle-ICP-MS†

**DOI:** 10.1039/d4ja00351a

**Published:** 2025-01-27

**Authors:** Lukas Brunnbauer, Laura Kronlachner, Elias Foisner, Andreas Limbeck

**Affiliations:** a TU Wien, Institute of Chemical Technologies and Analytics Getreidemarkt 9/164 1060 Vienna Austria lukas.brunnbauer@tuwien.ac.at andreas.limbeck@tuwien.ac.at

## Abstract

The need to analyze and characterize microplastics (MPs) is ever-increasing to monitor and understand their environmental impact. In this work, a developed calibration approach that utilizes an in-house-created polystyrene (PS) thin film for the sizing of MPs is presented, circumventing the need for certified particulate standard material. LA was used for sampling and transporting intact MPs of different sizes and polymer types to the ICP-MS. For the calibration, defined amounts of carbon were introduced into the ICP-MS by quantitatively ablating a polymer thin film with different laser spot sizes. With this approach, a LOD of 4.85 pg carbon was obtained, which translates to a size of 2.12 μm for spheric PS particles. The calibration using PS thin film was successfully applied to sampled PS MPs and allowed accurate sizing of 2 μm, 3 μm, and 4.5 μm particles. When using the PS calibration for determining polyvinyl chloride (PVC) and poly(methyl methacrylate) (PMMA) particle sizes, a good estimate of the size could be achieved despite the different compositions of the polymers. This indicates the universal applicability of the presented approach. The investigation of the transport efficiency showed that it is mainly influenced by particle size, and factors such as the polymer type and length of the transport line and carrier gas. Under optimum conditions, up to 95% of the sampled particles were detected.

## Abstract 2

This work demonstrates and investigates a calibration approach for sizing microplastic particles using single particle ICP-MS with laser ablation sampling circumventing the need for particulate standard material.

## Graphical abstract

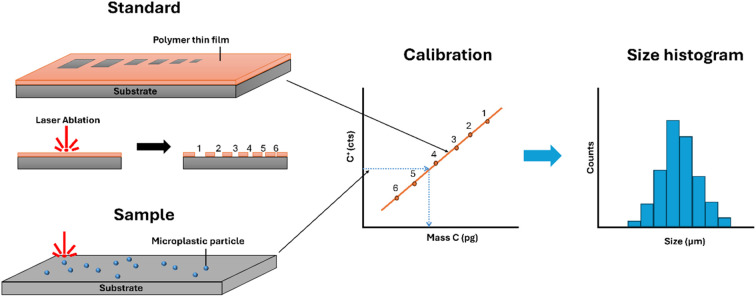

## Introduction

Plastics are omnipresent in almost all aspects of our modern society and have significantly contributed to our current standard of living due to their easy-to-tune properties. Therefore, plastics production is at an all-time high. Despite efforts to implement proper recycling and waste management strategies in the last few years, a significant fraction of plastic waste still ends up in the environment where, over time, microplastics (MPs) are formed.^1,2^ To study and understand the impact MPs have on our ecosystems and to be able to establish adequate regulatory standards, the need for a comprehensive characterization of MPs is arising. MPs characterization involves a multitude of different analytical techniques, including optical and electron microscopy,^3,4^ FT-IR and Raman spectroscopy,^5–7^ and liquid ICP-OES/MS combined with leaching protocols.^8–10^

One of the main questions for particle analysis is the aspect of particle size and size distribution, which can be addressed using single particle (sp)-ICP-MS, enabling the direct analysis of particle suspensions. In this case, each particle entering the ICP-MS generates a spike in the transient signal, which is evaluated. To obtain a particle size and number concentration of the analyzed suspension, there are two main requirements: a calibration is necessary to calculate the mass of each particle based on the signal it generated, and the transport efficiency (TE) must be known to estimate the number concentration correctly. Sp-ICP-MS has already been applied in the field of MPs analysis by several groups.^11–15^ The significant advantage is that a large number of particles can be measured in a short time, and if an ICP-ToF-MS is used, additional information about the (trace) metal composition of each particle is available.

Besides using sp-ICP-MS to analyze liquid samples, it was also demonstrated that the same concept can be combined with laser ablation (LA). In this case, the particles under investigation are sampled non-destructively by the LA system and transported to the ICP-MS.^16,17^ Using the combination of LA as a sampling method with sp-ICP-MS introduces vital benefits to the analysis approach, such as the high transport efficiency^18^ and the direct solid sampling, enabling imaging of the MP distribution in environmental, biological or medical samples.

First works in this field showed the application of LA-sp-ICP-MS for the analysis of MPs. Lockwood *et al.* proposed non-destructive sampling and analysis of MPs using LA-sp-ICP-MS by accurately determining the size of PS beads added to soil samples.^19^ Recently, van Acker *et al.* reported the analysis of different sized MPs by LA-sp-ICP-MS. Their work used low fluence laser pulses to non-destructively desorb MPs from a sample surface and transport them to an ICP-MS where each particle is detected in single-event mode. A linear relationship between known particle size and the obtained ^13^C^+^ signal was observed.^20^ Nevertheless, there are still limited routine quantification protocols available.

In this work, we develop a novel calibration strategy to enable the reporting of particle size distributions of MPs by non-destructive LA-sp-ICP-MS. To convert the ^13^C^+^ signal obtained from an individual particle to the particle size, the mass of carbon must be determined. Therefore, the application of standards is required. Recently, Vonderach *et al.*^21^ proposed using a sophisticated microdroplet generator to introduce well-defined masses of carbon to the ICP-MS, which can be used as standards for quantification in conventional liquid sp-ICP-MS of MPs. In our work, we propose a more universal approach for the calibration of LA-sp-ICP-MS without the need for particulate certified reference material. By quantitively ablating a well-defined in-house prepared PS-based polymer thin film with different laser spot sizes, we can uncomplicatedly and quickly vary the amount of carbon introduced to the ICP-MS. With the developed procedure, we can use the matrix-matched calibration to correctly determine the particle size distribution of PS microspheres. Additionally, we investigate the applicability of the calibration approach to other polymer types by analyzing PMMA and PVC microspheres. In a final step, we report transport efficiencies for different particle sizes of different polymer types by non-destructive LA-sp-ICP-MS analysis, which is a prerequisite for analyzing the number concentration of MPs.

## Experimental

### Chemicals and sample materials

The carrier gas used to constantly flush the laser chamber and transport the ablated material to the ICP-MS was helium (purity 99.999%). Helium with the same specifications was also used as collision gas in the ICP-MS in the kinetic energy discrimination (KED) mode. Argon (purity 99.999%) was used for the ICP-MS and as the make-up gas. The gases were obtained from Messer Austria (Austria).

Polystyrene flakes (atactic) from Thermo Scientific (Waltham, MA, USA) were used to create a standard thin film. For the preparation of the polymer solution, toluene (puriss. P.a., ACS reagent, ≥ 99.7% (GC)) obtained from Sigma Aldrich (St. Louis, MO, USA) was used as the solvent.

Microplastic samples in the form of 2.5% (w/v) polystyrene (PS) beads in aqueous suspension were purchased from Polysciences (Warrington, PA). The polybead® microspheres had nominal diameters of 2.00 μm, 3.00 μm and 4.50 μm with certified analyzed diameters of 2.07 μm, 3.10 μm and 4.78 μm respectively. The coefficient of variation is expressed as the SD/D ×100, with SD being the standard deviation and D being the actual diameter and has a value of <5% for the two smaller diameters and <7% for the bigger particles. The particles thus have a maximum standard deviation of ±0.10 μm, ±0.16 μm and ±0.33 μm in ascending order of diameter. Polyvinyl chloride (PVC) (5 ± 1 μm) and poly(methyl methacrylate) (PMMA) (2.96 ± 0.1 μm) microspheres were obtained from Lab261 (Palo Alto, CA) as 1% (wt/v) aqueous suspensions.

The suspensions were diluted using ethanol (abs. 100% a.r.) from Chem-Lab (Zedelgem, Belgium).

Microscope glass slides purchased from Marienfeld Superior (Lauda-Königshofen, Germany) were used as substrate for the samples and silicon wafers obtained from Infineon (Villach, Austria) were used as substrates for the standards.

### Preparation of a polystyrene (PS) thin film used as standards

Polystyrene flakes were dissolved in toluene to create a 5% (w/w) polymer solution. A 1 cm × 1 cm silicon wafer was placed in the SPC8-HC spin coater from Mendel Chemicals SRL (Chişinău, Moldova) and rotated at 4200 rpm at room temperature. 70 μL of the polystyrene solution was pipetted onto the spinning wafer, and the rotation speed was held constant for 90 seconds. The solvent evaporated completely during the spin coating process, resulting in a uniform polystyrene film with a thickness of 150 nm, which was determined using a Dektak XT stylus profilometer (Bruker Corporation, MA, USA). The variations in the thickness in the area used for the analysis (2 mm^2^) were below 5 nm. Using the silicon wafer as the substrate for the polymer film proved beneficial for the wetting of the surface and, thus, the uniformity of the film as compared to using microscopic glass slides as substrate material. With the above-described procedure, we were able to reproducibly prepare PS thin films with a defined thickness.

### Preparation of microspheres on a glass substrate

The polystyrene bead suspensions were diluted by factor 100 using ethanol, which resulted in a content of 5 × 10^7^ particles per mL to 5 × 10^6^ particles per mL. PVC and PMMA were treated the same way resulting in suspensions with 6 × 10^6^ particles per mL (PMMA) and 1 × 10^6^ particles per mL (PVC).

The microscope glass slides were cut into approximately 1 cm × 1 cm pieces for easier handling and cleaned thoroughly using ethanol. The glass substrate pieces were dried using nitrogen flow and placed in a drying oven heated to 80 °C for 5 minutes. 15 μL of the respective diluted microsphere suspension was pipetted onto the substrate, which was then placed back in the drying oven for another 90 s. With the optimized sample preparation, we still observe a coffee-ring effect with a significant number of MPs agglomerates on the edge of the droplet. Nevertheless, the optimization resulted in creating individual microspheres, largely avoiding agglomeration of the particles with approximately 300 particles per mm^2^ in the center of the sample, which is suitable for the subsequent LA-sp-ICP-MS analysis. Pictures of the sample surface, as observed through the LA microscope, can be found in the ESI (Fig. S1–S3).†

### LA-sp-ICP-MS instrumentation

LA-sp-ICP-MS analysis was carried out using an ImageGEO193 (ESL, Bozeman, Montana, US) equipped with an ArF Excimer laser emitting 193 nm. The system is equipped with the imaging cup in combination with a TV3 ultrafast washout cell. The LA system is connected to a Nexion5000 ICP-MS (PerkinElmer, Waltham, Massachusetts, US) using a PEEK capillary with an inner diameter of 0.03′′ with a dual concentric injector (DCI) (ESL, Bozeman, Montana, US). He is used as a carrier gas. The flows (Sniffer and chamber He) were optimized for the fastest washout when ablating the polymer standards. Ar is added to the He stream right before the ICP in the DCI to avoid peak broadening. The ICP-MS was tuned daily for maximum ^11^B^+^ intensity while keeping ^232^Th^16^O^+^/^232^Th^+^ below 1% when ablating the NIST612 glass standard (Standard Reference Material, National Institute of Standards and Technology, Gaithersburg, MD). ^11^B^+^ was selected to tune the sensitivity since it is the element present in NIST612, which has similar ionization energy and mass compared to ^13^C^+^, which is the analyte of interest in this work. Prior to LA-sp-ICP-MS analysis, a conventional dual detector calibration was carried out in liquid mode for proper calibration of the pulse counting and analog mode and for the determination of the dead time of the detector (35 ns). Syngistix 3.5 (Nanomodule 3.5) was used to record the ICP-MS data. An overview of measurement parameters is provided in Table S1 in the ESI.†

The laser ablation of the microspheres was conducted with a laser fluence of 1.4 J cm^−2^. Square 15 μm × 15 μm laser spots were placed onto the individual particles on the glass substrate. The particles were then sampled with single shots and transported to the ICP-MS.

For the calibration, the polymer thin film was quantitively ablated using different laser spot sizes ranging from 10 μm × 10 μm and 21 μm × 21 μm, resulting in different absolute masses of carbon being introduced to the ICP-MS. For each of the applied laser spot sizes, the ablation was repeated 20 times. The precise sizes and depths of the laser craters were determined using a profilometer to determine the ablated mass of carbon.

### Data evaluation

Data evaluation was carried out using a custom Jupyter notebook. In short, raw ICP-MS signals were imported and visualized using pandas and plotly. Peaks originating from either the ablation of the polymer thin film standards or the MPs introduced to the ICP-MS were detected using the scipy.signal.find_peaks function. Therefore, the parameters threshold, distance and width were used. Integration ranges for each peak were set by defining a window left and right from the peak center. Even though the peak width of signals originating from the ablation of the standards differs significantly from the peak width of MPs, an integration window of the same size was used for both to account for the background stemming mainly from the atmospheric CO_2_ as well as different components of the LA system (see Fig. 2(b) and (d)). Finally, the data points in the integration window are summed. Slope and intercept of the calibration function are calculated using scipy.stats.linregr.

## Results and discussion

This work aims to determine the particle size distributions of MPs by non-destructive LA-sp-ICP-MS analysis while monitoring ^13^C^+^. For that, we propose using a PS-based polymer thin film: by quantitative ablation of the thin film with different laser spot sizes, we can introduce well-defined carbon masses to the ICP-MS, resulting in a calibration function. With the established calibration function we can accurately determine the mass of an individual particle based on the spike generated in the transient ICP-MS signal. The particle's diameter can be calculated by assuming spherical particles with known density and carbon content.

## Optimization of measurement parameters

### Sample preparation

In a first step, the preparation of MPs on a glass substrate was optimized with the goal of avoiding agglomeration to be able to sample individual MPs. When optimizing the solvent used for diluting the MPs suspensions, dilution with ultrapure water was unfavorable for avoiding agglomeration of particles, while dilution with ethanol aided in generating non-agglomerated particles. The substrate on which MPs were deposited also influenced the agglomeration behavior of the particles: polished silicon wafers proved to be disadvantageous for the desired result, whereas microscope glass slides were beneficial for avoiding agglomeration. Lastly, depositing on heated (80 °C) and non-heated substrates was compared, which showed that the quicker evaporation on the heated substrate is beneficial for creating dispersed, non-agglomerated microsphere samples in the center of the sample.

### Laser ablation settings

There are two main parameters on the LA side to consider for optimization: He gas flows and laser energy.

The He gas flows mainly affect the washout of the ablated material from the standards and the transport efficiency of the MPs to the ICP-MS, as well as the level of the carbon background. As the particle size distribution after an ablation event using an excimer laser is typically in the nm range,^22,23^ whereas the MPs we want to transport to the ICP-MS are in the μm range, He gas flows can only be optimized for one of the two cases.

We optimized the measurement conditions to get a transient signal as short as possible for the ablation of the PS thin film, resulting in optimized sensitivity for the detection of the standards. Both He gas flows were tested in a range of 100 to 500 mL min^−1^ with 50 mL min^−1^ steps in all possible combinations to achieve the shortest possible peak width upon ablating the film standards. The resulting optimized He flows were 200 mL min^−1^ for the chamber flow and 250 mL min^−1^ for the sniffer flow.

For our experiments we must ensure quantitative ablation of the polymer thin film for reliable use as a standard. On the other hand, we want to use a laser fluence as low as possible to avoid the formation of gaseous carbon species, resulting in a satellite peak in the transient signal 2,^24^ which will be discussed in more detail in the next section. Additionally, using a low laser fluence ensures the intact sampling of individual MPs^20^ and limits the formation of gaseous carbon after LA. Therefore, the laser energy is set to the minimum value where we can ensure quantitative ablation of the polymer thin film. Laser fluences of 0.6 J cm^−2^ to 2 J cm^−2^ were tested in steps of 0.2 J cm^−2^. In our case, the optimal laser fluence to meet these requirements was 1.4 J cm^−2^, where we observed complete quantitative ablation of the polymer film and could still guarantee non-destructive sampling of the particles. Using this fluence, we also confirmed that no ablation of the Si-substrate occurs by monitoring ^29^Si^+^ in preliminary experiments.

### Transient signals

**Fig. 1:**
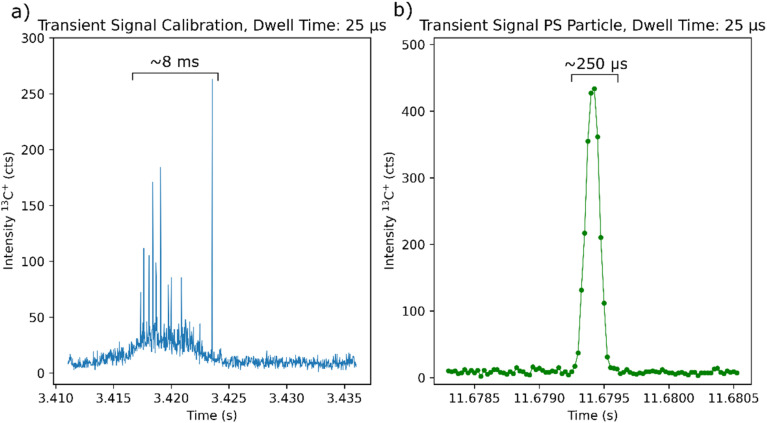
Exemplary transient signals with the shortest dwell time possible (25 μs) for (a) ablation of the polymer thin film and (b) for the sampling of an individual MP.

In the next step, we investigated the transient signals obtained at the ICP-MS after ablating the prepared PS thin film used for the calibration, compared to the signals obtained by sampling individual MPs. Using the shortest dwell time possible (25 μs) enables us to fully capture the evolution of the transient signals confirming the transport of intact MPs to the ICP-MS based on the peak width (250 μs) (Fig. 1(b)). Typically, conventional LA-ICP-MS signals are not detected with such a high time resolution. Therefore, this allows us to get deeper insights into the particle aerosol generated after the ablation event: the aerosol generated after ablating the PS thin film results in a peak with a width of approximately 8 ms and most likely reaches the ICP-MS the form of individual nanoparticles^23^ and agglomerates indicated by the increase of the signal and the spikes observed (Fig. 1(a)).

An important aspect to consider is the different timescales in the transient signals from ablating the PS thin film and introducing an individual MP to the ICP-MS. This is because the polymer thin film forms a sample aerosol upon ablation while the MP remains intact when sampled. As shown in Fig. 1 with state-of-the-art fast washout cells, a peak width of a regular ablation event in the range of a few ms can be expected, whereas introducing an individual MP results in a signal in the range of a few hundred μs.

In theory, the integrated signal of a MP particle with a certain mass of carbon is the same as integrating the signal originating from ablating the same mass of carbon from a polymer thin film. Nevertheless, due to the difference in peak widths, the MP will result in a narrow peak with a high peak maximum, whereas the ablation event of the standard will result in a broad peak with a lower peak maximum, resulting in a difference in the signal to background ratios.

Potential deviations from the theoretical expectations in the experiment could be caused by differences in the ablation efficiency between the polymer film and particles, as well as different detector responses to the varying peak width.

A problem to consider is the two different measurement modes of typical detectors used in ICP-MS (pulse counting and analog mode). Due to the difference in peak maxima caused either by different sized MPs or by the different peak width when ablating the standard, different detector modes may be used to record parts of the signals. Typically, this is not a problem if an adequate detector cross-calibration is established. Nevertheless, for reasons not known, it was not possible to apply proper corrections when recording data with ultra-short dwell times in the nanomodule of the instrument. We observed this by a steepening of the calibration curve for higher masses of carbon, which could not be explained by factors such as plasma load or ionization efficiency in the ICP.

Consequently, we need to keep the signal maximum of the particles (and also the peak maxima of the standards) below the threshold of the detector recording the signal in the analog mode. To achieve this, we used a KED gas flow to reduce the signal, similar to van Acker *et al.*^20^ A KED gas flow of 0.6 mL min^−1^ provided sufficient signal suppression regarding those considerations.

Nevertheless, it should be noted that this approach decreases the sensitivity of the method and is only necessary because of inadequate detector cross-calibration. However, by decreasing the sensitivity, the method's applicability for samples consisting of MP particles with smaller carbon content (*e.g.*, smaller-sized particles) is limited.

**Fig. 2:**
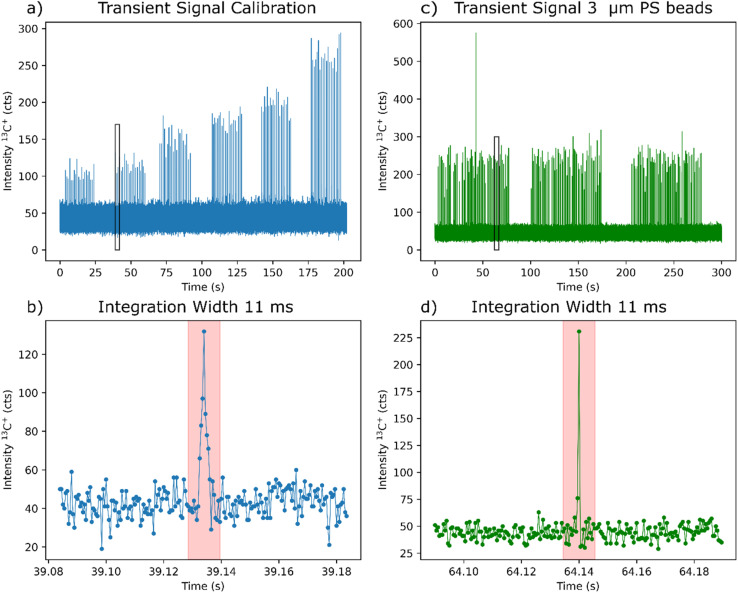
Transient signals for ^13^C^+^ using a laser fluence of 1.4 J cm^−2^, dwell time of 500 μs and a KED gas flow of 0.6 mL min^−1^ for the calibration standards (a) and the for the 3 μm PS particles exemplarily (c) with corresponding zoomed figures (b) and (d) showing the black framed rectangles in the top figures enlarged.

Additionally, we increased the dwell time to 500 μs, which resulted in a well-defined peak for the ablation of the standard (see Fig. 2(b)). Therefore, the integration ranges can be set more precisely. The selected dwell time is still adequate for monitoring the individual MPs events see (Fig. 2(d)), which are in the timescale of 250–500 μs.^25^

## Calibration

The calibration approach is based on ablating a PS-based polymer thin film with different laser spot sizes. This leads to different precisely defined amounts of polymer being ablated and, subsequently, different amounts of carbon being introduced to the ICP-MS. The optimized KED gas flow, as described above, was used for the analysis. Further measurement parameters are given in Table S1 in the ESI.†

The laser energy was optimized to the minimum energy in the preliminary experiments, resulting in a quantitative ablation. It is known from previous works that in LA-ICP-MS, typically, two-phase transport occurs for carbon. van Helden *et al.*^24^ demonstrated that the amount of particulate and gaseous carbon formed after an ablation event depends on laser energy. With increasing laser energy, the fraction of gaseous carbon increases. Since the formed particulate carbon yields a much faster washout and sharper peaks at the ICP-MS compared to the formed gaseous carbon, this is preferred to increase the sensitivity. Fig. 2(a) and (b) show the transient signal for the calibration with a zoomed-in depiction. The peak in the signal stemming from the particulate carbon appears between 39.12 s and 39.14 s. Experiments with higher laser fluences show that the gaseous carbon peak would appear between 39.16 s and 39.18 s. With our optimized measurement conditions, we do not observe a significant gaseous carbon peak when analyzing the PS thin film, and we can only evaluate the particulate peak.

Six different laser spot sizes (*n* = 20 for each spot size) were used to ablate the PS-based polymer thin film with the optimized settings to introduce different amounts of carbon to the ICP-MS (Fig. 2(a)). Fig. 2(b) depicts one peak of the standard's measurement, which introduces a similar amount of carbon into the ICP-MS as the 3 μm PS particles (Fig. 2(d)). This illustrates the difference in the peak widths and heights generated by a similar amount of carbon reaching the ICP-MS as a sample aerosol generated by the ablation of the standard polymer thin film or an intact polymer particle from the MP sample.

Six calibration points were created by ablating the defined amount of the PS-based thin film, whereas the first point of the calibration, representing 0 pg of introduced carbon, was created by integrating the background signal. For that purpose, 20 randomly selected positions within the background signal were integrated. Thus, each of the seven calibration points represents the mean of 20 integration values and shows their corresponding standard deviation as error bars.

**Fig. 3:**
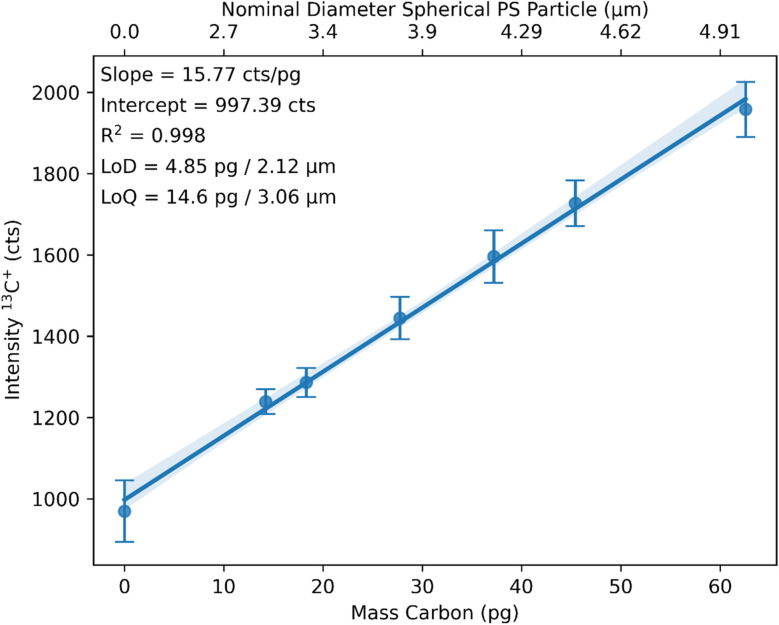
Generated linear calibration function showing the relation between the intensity for the ^13^C^+^ signal and the mass of carbon (lower *x*-axis) and, respectively, the nominal diameter of a spherical PS particle (upper *x*-axis).

The integrated peak areas of the calibration signal are now plotted against the mass of carbon in pg to build a calibration function (Fig. 3). The mass of carbon is calculated based on profilometer measurements (area and depth) of the craters and considering the density and carbon content of PS. A second *x*-axis is added in Fig. 3 to illustrate the relationship between the mass of carbon and the equivalent diameter of a spherical PS particle.

The calibration showed an *R*^2^ value of 0.998, a limit of detection (LoD) of 4.85 pg or 2.12 μm nominal PS diameter, and a limit of quantification (LoQ) of 14.6 pg or 3.06 μm nominal PS diameter. LoD and LoQ were calculated based on the noise of the gasblank. The obtained LoD for carbon is comparable to the LoD of 4.83 pg reported by Vonderach *et al.*^21^ using a microdroplet approach for the calibration.

## Particle analysis

### Nominal diameters of PS MPs

The position of individual PS MPs on the substrate was determined with the camera in the LA system. Each particle was sampled by an individual laser shot right on the particle's position. Using this approach compared to scanning large areas of the sample with parallel line scans allowed us to avoid multi-events where multiple particles enter the ICP-MS quasi simultaneously. Additionally, with this approach, the transport efficiency is improved since particles tend to be displaced by the shockwave of a close-by laser shot without entering the transfer line to the ICP-MS, which occurs frequently for line scans.

The spike signals generated by the analysis of the MPs particles (shown exemplarily for 3 μm PS particles in Fig. 2(c) and (d)) were integrated, and the calibration function was used to calculate the mass of the analyzed PS beads. The nominal diameter of the different-sized particles was calculated under the assumption that all the PS particles were perfectly spherical.

**Fig. 4:**
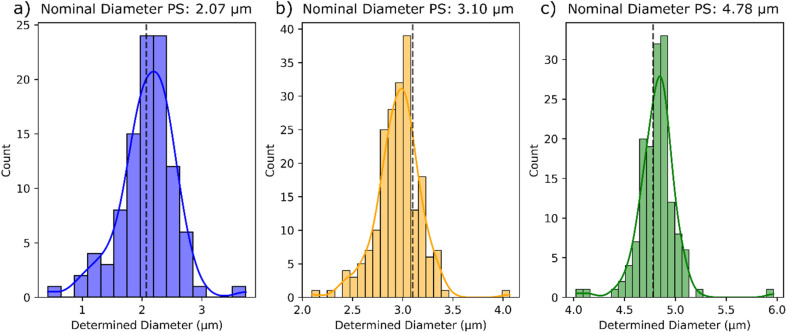
Size distribution of the evaluated PS microplastic particles for the 2.07 μm particles (a), the 3.10 μm particles (b), and the 4.78 μm particles (c), including a Kernel density estimation curve and the nominal certified diameters marked with the dotted black lines.

The resulting diameters for the detected particles are shown in Fig. 4 as size distribution histograms. The maximum of the histogram based on the kernel density function for the analyzed particles are 2.20 μm, 2.99 μm, and 4.85 μm, which is in good agreement with the certified values shown in dotted lines. The deviations from the nominal diameters are 6.3% for the 2.07 μm particles, 3.5% for the 3.10 μm particles, and 1.5% for the 4.78 μm particles.

It should be pointed out that the mass of the PS particles with a determined diameter of 2.2 μm is close to the LoD obtained from the calibration function, explaining the increased uncertainty represented by the broader distribution. Nevertheless, the determined particle sizes are in good agreement with the certified values demonstrating the applicability of the developed method over the full range of the calibration.

### Application of the calibration approach to other polymer types

To investigate the applicability of using a PS thin film for quantifying the size distribution of other polymer-type MPs, we analyzed PMMA and PVC microspheres with a nominal diameter of 3 μm and 5 μm, respectively. When applying a PS-based calibration to evaluate particles of different polymer types (PMMA, PVC), matrix effects are expected due to the difference in chemical composition, which can limit the applicability of the presented method as they cause divergent behavior of the samples and the standards.^26^ Compared to conventional LA-ICP-MS, where matrix effects occur in the ablation process, transport, and the ICP, in our case, matrix effects only in the ICP must be considered since intact particles are sampled. In the ICP, matrix effects mainly affect the atomization and ionization efficiency of the analyte of interest. Especially the Cl present in the ICP when introducing PVC particles may influence the ionization efficiency of carbon and, therefore, may suppress the signal. Considering the polymers' carbon content (PS: 92.3%, PMMA: 60.0%, and PVC: 38.7%) and density (PS: 1.05 g cm^−3^, PMMA: 1.18 g cm^−3^, and PVC: 1.38 g cm^−3^), other polymer types can be evaluated as well by the presented calibration approach.

**Fig. 5:**
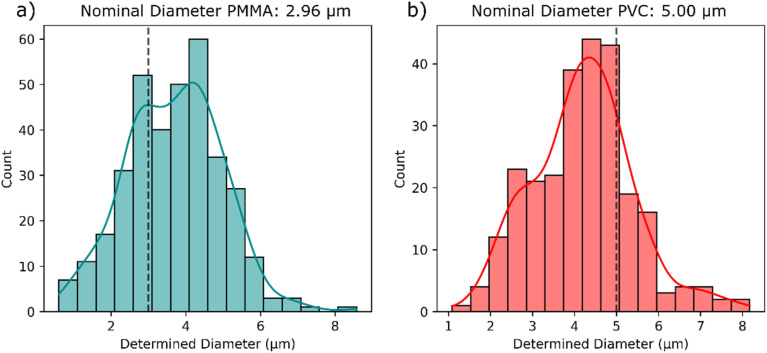
Size distribution of the evaluated microplastic particles for the 2.96 μm PMMA particles (a) and the 5 μm PVC particles (b), including a Kernel density estimation curve and the nominal certified diameters marked with the dotted black lines.

Particle size distributions obtained for PMMA and PVC are shown in Fig. 5(a) and (b). The obtained histograms show a maximum close to the reference value with a broader distribution compared to the PS particles. Even though the obtained results show some deviation from the reference value, it should be considered that a deviation of 50% in the obtained carbon mass of a 5 μm PVC particle only results in an error of 20% of the determined particle diameter. This means a 5 μm PVC particle is detected as a 4 μm PVC particle which is still a good estimate for the size of the particle, especially considering the non-matrix matched calibration. Despite the deviation, this can still be useful for assessing the size range of a microplastic sample.

Depending on the application, applying a non-matrix-matched calibration still results in reliable determination of MP sizes indicating the universal applicability of the developed calibration approach to other polymer types as well. This means that analysis of polymer types where no certified reference material is possible, in particular since most polymer types have a similar composition and, thus, a similar carbon content (with the exception of certain polymer types such as PVC and PTFE).

### Transport efficiency (TE)

**Fig. 6:**
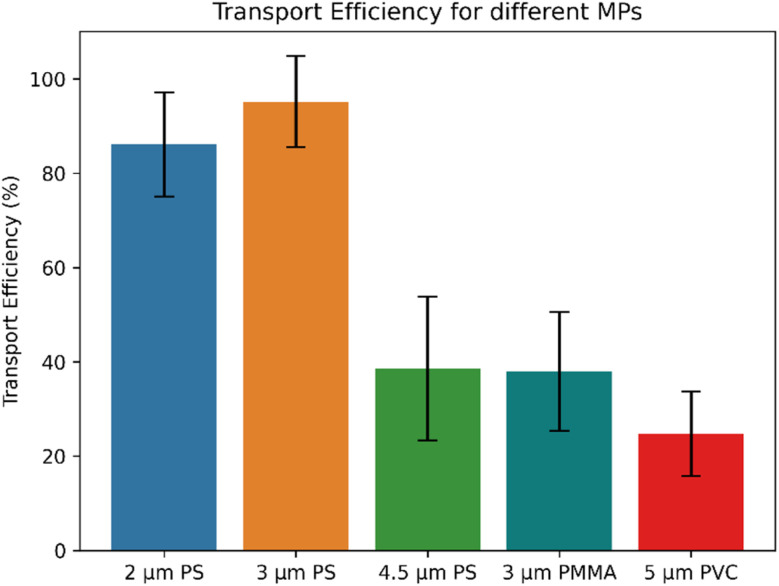
The transport efficiency (TE) in percent for the different MPs under investigation determined by sampling 50 particles 20 times.

When performing sp-ICP-MS, besides obtaining the correct particle size by applying a calibration, it is also of interest to determine the number concentration of the particles present in the sample under investigation. Therefore, it is crucial to determine the TE of the particles to be able to measure the number concentration accurately. In conventional liquid sp-ICP-MS analysis, the TE is typically determined by measuring a standard with a known number concentration of particles and comparing that to the actual number of particles detected when analyzing a known volume of the liquid. When using LA-ICP-MS for single particle analysis, no solid reference material with a known particle number concentration in a defined area is available. This is in contrast to liquid sp-ICP-MS, where liquid suspension certified reference materials are obtainable. In our case, the particles under investigation were visible in the microscope of the LA system (see ESI†). Therefore, we determined the TE by sampling a known number of individual particles (20 times 50 particles) and comparing it to the detected events in the transient ICP-MS signal. Transport efficiencies determined for the particles under investigation in this study are shown in Fig. 6. A significantly lower TE was observed for the large PS (4.5 μm) particles than the two smaller (2 μm and 3 μm) PS particles. This lower transport efficiency for larger particles means that for MPs above a certain size, a higher number of particles need to be sampled to gain a representative dataset. Additionally, we observed that the TE depends on the polymer type: for 3 μm PS particles, we observed a much higher TE compared to 3 μm PMMA particles. When considering the TE of the investigated particles, there are mainly three aspects to be considered:

• The particle must be successfully desorbed from the sample surface and introduced to the He stream. This is most likely more favorable for smaller particles. Additionally, successfully desorbing the particle and introducing it to the He stream may be influenced by the polymer type due to a different interaction of the particle with the substrate, and the different densities.

• The particle has to follow the He stream to the ICP-MS without colliding with the tubing walls, which would most likely cause the particle to get stuck in the tubing. Again, this is most likely more favorable for smaller particles.

• He gas flow is optimized for fast washout of the generated aerosol when ablating the polymer film standard, resulting in sharp peaks and not for the TE of individual MP particles.

## Conclusion

In this study, we present the determinations of particle sizes and size distributions of microplastics using sp-ICP-MS combined with laser ablation sampling. The developed methodology enabled the effective determination of the diameters of PS microplastic samples of different sizes (ranging from 2 to 4.5 μm) with deviations of ≤ 6.3% from the certified diameters without needing certified standard materials.

The utilized calibration method involves introducing defined amounts of carbon into the ICP-MS through laser ablation sampling. We achieved this by ablating a thin polystyrene film created by spin coating a polymer solution. By quantitatively ablating the polymer film with different laser spot sizes, we can accurately determine the amount of polymer and carbon reaching the ICP-MS by measuring the depths and areas of the laser craters. We created a calibration function by correlating the measured carbon signal intensities with the known introduced carbon content reaching detection limits (LoD) of 4.85 pg of carbon.

With this calibration, the mass of carbon of non-destructively ablated microplastic particles can be determined, and the size of the particles can be calculated using information about the geometry, density, and carbon content of the samples.

We also demonstrated the applicability of the approach for microplastic particles of different polymer types (*i.e.* PMMA and PVC), where a good estimate of the certified size could be determined when using the PS thin film as the calibration standard. These results indicate the general applicability of the approach.

Furthermore, we investigated the transport efficiencies for differently sized particles and different polymer types. We found that the transport efficiency is significantly influenced by particle size, with larger particles having lower transport efficiencies than smaller particles, as well as by the polymer type. Additionally, it can be assumed that other substantial factors affecting the transport efficiencies include the carrier gas flow and the properties of the transfer line.

## Data availability

The data supporting the findings of this study are available within the article or its ESI.† Other relevant data can be shared upon reasonable request.

## Conflicts of interest

The authors declare that they have no conflicts of interest.

## Supplementary Material

JA-040-D4JA00351A-s001

## References

[cit1] Koelmans B. , Pahl S., Backhaus T., Bessa F., van Calster G., Contzen N., Cronin R., Galloway T., Hart A. and Henderson L., A Scientific Perspective on Microplastics in Nature and Society, SAPEA, 2019

[cit2] Hale R. C., Seeley M. E., La Guardia M. J., Mai L., Zeng E. Y. (2020). J. Geophys. Res.:Oceans.

[cit3] Jung S., Cho S.-H., Kim K.-H., Kwon E. E. (2021). Chem. Eng. J..

[cit4] Desforges J.-P. W., Galbraith M., Dangerfield N., Ross P. S. (2014). Mar. Pollut. Bull..

[cit5] Käppler A., Fischer D., Oberbeckmann S., Schernewski G., Labrenz M., Eichhorn K.-J., Voit B. (2016). Anal. Bioanal. Chem..

[cit6] Xu J.-L., Thomas K. V., Luo Z., Gowen A. A. (2019). TrAC, Trends Anal. Chem..

[cit7] Araujo C. F., Nolasco M. M., Ribeiro A. M. P., Ribeiro-Claro P. J. A. (2018). Water Res..

[cit8] Turner A., Holmes L., Thompson R. C., Fisher A. S. (2020). Water Res..

[cit9] Holmes L. A., Turner A., Thompson R. C. (2012). Environ. Pollut..

[cit10] Massos A., Turner A. (2017). Environ. Pollut..

[cit11] Bolea-Fernandez E., Rua-Ibarz A., Velimirovic M., Tirez K., Vanhaecke F. (2020). J. Anal. At. Spectrom..

[cit12] de Vega R. G., Goyen S., Lockwood T. E., Doble P. A., Camp E. F., Clases D. (2021). Anal. Chim. Acta.

[cit13] Trujillo C., Pérez-Arantegui J., Lobinski R., Laborda F. (2023). Nanomaterials.

[cit14] Hendriks L., Mitrano D. M. (2023). Environ. Sci. Technol..

[cit15] Velimirovic M., Tirez K., Verstraelen S., Frijns E., Remy S., Koppen G., Rotander A., Bolea-Fernandez E., Vanhaecke F. (2021). J. Anal. At. Spectrom..

[cit16] Yamashita S., Yoshikuni Y., Obayashi H., Suzuki T., Green D., Hirata T. (2019). Anal. Chem..

[cit17] Metarapi D., Šala M., Vogel-Mikuš K., Šelih V. S., van Elteren J. T. (2019). Anal. Chem..

[cit18] Garcia C. C., Lindner H., Niemax K. (2007). Spectrochim. Acta, Part B.

[cit19] Lockwood T. E., de Vega R. G., Clases D. (2021). J. Anal. At. Spectrom..

[cit20] van Acker T., Rua-Ibarz A., Vanhaecke F., Bolea-Fernandez E. (2023). Anal. Chem..

[cit21] Vonderach T., Gundlach-Graham A., Günther D. (2024). Anal. Bioanal. Chem..

[cit22] Kuhn H.-R., Günther D. (2004). J. Anal. At. Spectrom..

[cit23] Hahn D. W., Ediger M. N., Pettit G. H. (1995). J. Appl. Phys..

[cit24] van Helden T., Mervič K., Nemet I., van Elteren J. T., Vanhaecke F., Rončević S., Šala M., Van Acker T. (2024). Anal. Chim. Acta.

[cit25] Montaño M. D., Olesik J. W., Barber A. G., Challis K., Ranville J. F. (2016). Anal. Bioanal. Chem..

[cit26] Bonta M., Limbeck A. (2018). J. Anal. At. Spectrom..

