# Supplementary material for: Novel calibration approach for particle size analysis of microplastics by laser ablation single particle-ICP-MS
Source: J Anal At Spectrom. 2025 Jan 27;40(3):753–61. doi: 10.1039/d4ja00351a (PMC11783204; doi:10.1039/d4ja00351a)
Supplement: JA-040-D4JA00351A-s001 [file JA-040-D4JA00351A-s001.pdf]

Supplementary Information (SI) for Journal of Analytical Atomic Spectrometry.
This journal is © The Royal Society of Chemistry 2025

Novel calibration approach for particle size analysis of microplastics by Laser Ablation Single Particle-ICP-MS

Lukas Brunnbauer *[1], Laura Kronlachner *[1], Elias Foisner[1] and Andreas Limbeck[1]

[1]TU Wien, Institute of Chemical Technologies and Analytics, Getreidemarkt 9/164, 1060 Vienna, Austria

* These authors contributed equally to this work.

Table S1: LA and ICP-MS measurement parameters

ImageGEO193		Nexion5000	
Laser Fluence (J/cm^2)	1.4	RF power (W)	1600
Spot size (square shape) (µm)	15 x 15 for MPs 10 x 10 , 12 x 12, 14 x 14, 16 x 16, 18 x 18, 20 x 20 for standards	Ar make-up gas (L/min)	1.25
Chamber He flow (ml/min)	200	Cool-gas flow (L/min)	16
Sniffer He flow (ml/min)	250	Detected Isotopes	$^{13}C^{+}$
Shots per position	1	Dwell time (µs)	500

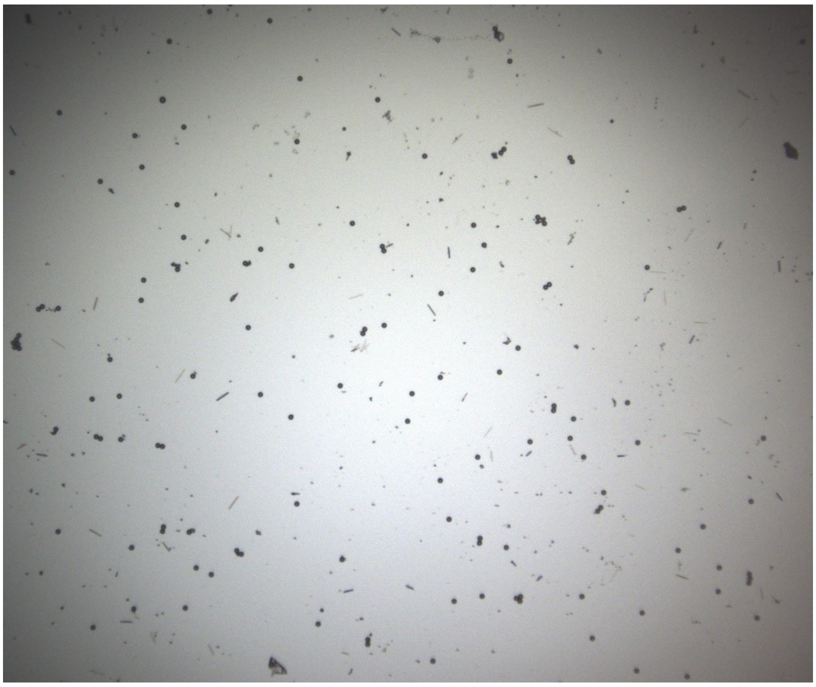

Figure S1: PS 4.5 µm particles on the substrate

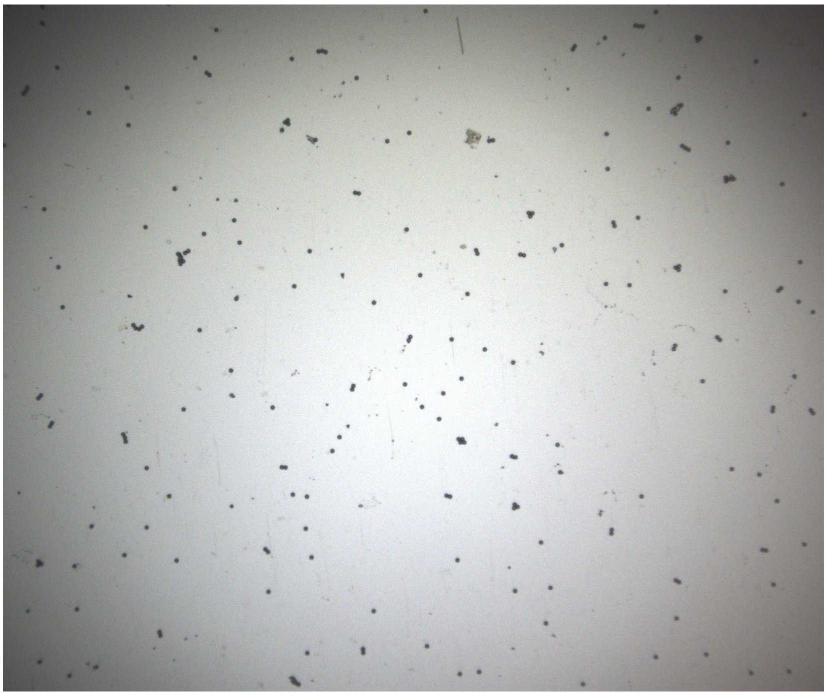

Figure S2: PS 3 µm particles on the substrate

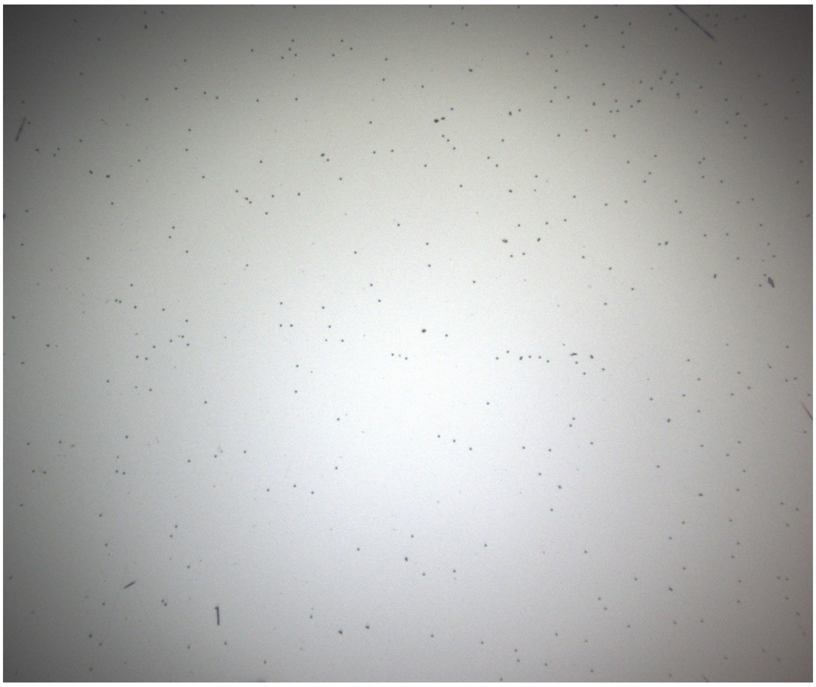

Figure S3: PS 2 µm particles on the substrate
